# Mumps-Related Disease Burden in Japan: Analysis of JMDC Health Insurance Reimbursement Data for 2005–2017

**DOI:** 10.2188/jea.JE20200048

**Published:** 2021-08-05

**Authors:** Satoko Ohfuji, Akira Takagi, Takashi Nakano, Hideaki Kumihashi, Munehide Kano, Toshihiro Tanaka

**Affiliations:** 1 Department of Public Health, Osaka City University Graduate School of Medicine; and Research Center for Infectious Disease Sciences, Osaka City University Graduate School of Medicine, Osaka, Japan; 2 Department of Otorhinolaryngology, Head and Neck Surgery, Shizuoka General Hospital, Shizuoka, Japan; 3 Department of Pediatrics, Kawasaki Medical School, Okayama, Japan; 4 Global Vaccine Business Unit, Takeda Pharmaceutical Company Limited, Tokyo, Japan; 5 Department of Pediatrics, Shizuoka Kosei Hospital, Shizuoka, Japan

**Keywords:** disease burden, mumps, mumps complication

## Abstract

**Background:**

Mumps vaccination coverage is low in Japan, partly because of its voluntary nature. Although pediatric cases of mumps virus infection are captured by the National Epidemiological Surveillance of Infectious Diseases program under the Infectious Disease Law, there are currently no data regarding the occurrence of mumps and its complications in adults.

**Methods:**

We investigated the annual incidence rates of mumps and its complications based on health insurance reimbursement data for 5,209,660 individuals aged 0–64 years for 2005–2017, obtained from JMDC Inc., to estimate the mumps-related disease burden during this period.

**Results:**

There were three mumps outbreaks (2006, 2010, and 2016) during 2005–2017. The annual incidence of mumps was highest in individuals aged 0–5 years (808–3,792 per 100,000 persons), followed by those aged 6–15 years (658–2,141 per 100,000 persons). The incidence of mumps was higher in females than in males (male/female ratio, 0.90). Among mumps-related complications, the overall incidence (per 1,000 mumps cases) was highest for orchitis (6.6), followed by meningitis (5.8), deafness (1.3), pancreatitis (0.5), and encephalitis (0.3). No cases of oophoritis were noted. The overall incidence of mumps-related complications was 2.5 times higher in males than in females.

**Conclusions:**

This study revealed the disease burden due to mumps and its complications in Japan during 2005–2017. These data suggest the need for mumps-prevention measures in adolescents and adults, as well as in children.

## INTRODUCTION

Mumps is a viral infection primarily affecting children, causing inflammation of the parotid gland, potentially leading to serious complications, such as orchitis, meningitis, encephalitis, and pancreatitis.^1^ Live attenuated mumps vaccines were developed in the 1960s,^2^^–^^4^ and mumps vaccines have recently been introduced into the routine pediatric immunization program in more than 120 countries,^5^ resulting in a marked reduction in mumps outbreaks. However, the routine immunization program in Japan does not currently include mumps vaccines.

Mumps vaccination in Japan was started in 1981 but the vaccination rate was low because of the voluntary nature of the vaccination, with major outbreaks every 3 to 5 years.^6^ Measles-mumps-rubella (MMR) vaccines were introduced into the routine immunization program in April 1989, resulting in an increased vaccination rate and decrease in reported mumps cases. However, MMR vaccines were discontinued in April 1993 because of reports of aseptic meningitis caused by the mumps vaccine element of the MMR vaccines.^7^ Monovalent mumps vaccines have since been used for vaccination on a voluntary basis, but the vaccination coverage has been as low as 20–30%, with large-scale mumps outbreaks repeated every 4 or 5 years. In contrast, the reported vaccination coverages in other developed countries are as high as 90% and above,^6^^,^^8^ which is associated with reduced incidence rates of mumps. For example, the incidence of mumps in the United States in 2011 was less than one-hundredth of that in Japan.^9^

Japan has a National Epidemiological Surveillance of Infectious Diseases program under the Infectious Disease Law, in which designated pediatric sentinel sites are required to report the numbers of mumps cases weekly to provide surveillance of mumps outbreaks in the pediatric population.^10^ However, outbreaks of mumps and the occurrence of mumps-related complications among adults remain unknown. With this background, we investigated the incidence rates of mumps and its complications in people aged 0–64 years in Japan by analyzing a health insurance reimbursement database obtained from JMDC Inc.^11^ (hereinafter referred to as the JMDC Database).

## METHODS

### Study design and data

This was a retrospective observational study using the JMDC Database, which stores health insurance reimbursement data in Japan, together with healthcare service data on beneficiaries collected from multiple health insurance unions since 2005. The database covered approximately 4% of the entire Japanese population. Health insurance reimbursement data were entered using the International Classification of Diseases Version 10 (ICD-10) codes. This study analyzed the data for the period from January 2005 to December 2017. The dataset contained each patient’s de-identified ID number, sex, birth year and month, diagnosis (ICD-10 code) according to the physician who examined the patient, and the date of diagnosis. Data extraction, program construction, and data analysis were conducted independently by two researchers. Quality assurance was achieved by complete agreement of the obtained results between the two researchers.

### Analysis population and follow-up investigation

The observation period for each patient was defined as the period of documented subscription to the health insurance policy between January 2005 and December 2017. The observation period in each year was the period of documented subscription to the health insurance policy in that year. The analysis population was defined as all persons aged 0–64 years with documented subscription to the health insurance policy for at least 3 months, excluding those with recurrent parotitis defined by meeting all the following criteria: 1) mumps diagnosed during the observation period; 2) no mumps complications diagnosed during the observation period; 3) parotitis diagnosed within 6 months before a diagnosis of mumps during the observation period; and 4) uncomplicated mumps or uncomplicated parotitis diagnosed within 2–6 months after a diagnosis of mumps during the observation period. Cases with recurrent parotitis were excluded because the inclusion of such cases would interfere with the proper evaluation of mumps-related diseases.

The inclusion of subjects with short observation periods might have resulted in selection bias when calculating the annual incidence. To minimize the risk of such a bias, the analysis of annual incidence excluded people who completed the observation period in or before February of the year and people who started the observation period in or after November of the year, unless they were 0-year-old infants.

### Definitions of mumps diagnosis and time of onset

In the JMDC Database, mumps cases were defined as patients with an ICD-10 code of ‘B26 (Mumps)’, entered in the disease section for health insurance reimbursement. The time of mumps onset was determined based on the treatment start date recorded in the disease section for health insurance imbursement. The year and month of onset used the earliest treatment start date among the data recorded for the disease diagnosed in the observation period.

### Definitions of mumps-related complications and their occurrence

Complications of mumps were defined as any of the following ICD-10 subcodes of mumps-related complications, recorded in or after the month of mumps onset: mumps orchitis (B26.0), mumps meningitis (B26.1), mumps encephalitis (including myelitis, encephalitis, and encephalomyelitis) (B26.2), mumps pancreatitis (B26.3), mumps deafness (B26.8), mumps myocarditis (B26.8), mumps nephritis (B26.8), mumps oophoritis (B26.8), mumps polyneuropathy (B26.8), mumps hepatitis (B26.8), and mumps arthritis (B26.8). The calculation of the incidence rates of mumps-related complications excluded people who received a diagnosis of any of the following within 3 months of subscription of the health insurance policy, because the infection could have started before the observation period: mumps, mumps orchitis, mumps meningitis, mumps encephalitis (mumps myelitis, encephalitis mumps, mumps encephalomyelitis), mumps pancreatitis, mumps arthritis, mumps myocarditis, mumps nephritis, mumps polyneuropathy, mumps hepatitis, mumps oophoritis, and other mumps-related complications. Calculation of the incidence of mumps deafness excluded people with a diagnosis of congenital deafness. Calculation of the incidence of mumps orchitis excluded females.

The interval from mumps onset to the diagnosis of a mumps-related complication was disregarded in the analysis. The year and month of the onset of a mumps-related complication was regarded as the year and month of the onset of mumps.

### Statistical analysis

The annual incidence rate of mumps in people aged 0 years (per 100,000 person-years) was calculated as ‘Number of people with mumps/Number of analyzed persons × 100,000’. For people aged ≥1 year, the annual incidence rate of mumps (per 100,000 person-years) was calculated as ‘Number of people with mumps/Number of analyzed persons/(Number of months of observation in the analyzed persons/12) × 100,000’. The annual incidence was calculated for the overall population and also by age group (0–5 years, 6–15 years, 16–25 years, 26–35 years, 36–45 years, 46–55 years, and 56–64 years). The most prevalent age group for mumps is 0–5 years old, and we therefore analyzed this specific age group and subsequent 10-year age groups. In addition, the overall incidence rate of mumps during the study period was calculated for the overall population, and according to age group and sex.

The annual incidence of mumps-related complications (per 1,000 mumps cases) was calculated as ‘Number of people with mumps-related complication/Number of people with mumps × 1,000’. However, the annual incidence of orchitis (per 1,000 mumps cases) was only calculated in male cases, as ‘Number of people with mumps orchitis/Number of males with mumps × 1,000’. The annual incidence was calculated for the overall population and also by age group. In addition, the overall incidence rate of mumps-related complications during the study period was also calculated for the overall population, and according to age group and sex.

Confidence intervals (CIs) for the annual incidence were calculated using the Clopper–Pearson method. For the overall incidence across years, an approximate 95% CI based on a Poisson distribution was calculated.

All statistical analyses were conducted using the statistical analysis software SAS version 9.4 (SAS Institute Japan Limited, Tokyo, Japan). Sex differences were assessed using χ^2^ tests with a significance level of 0.1%.

### Ethical approval and informed consent

The Japanese Ethical Guidelines for Medical and Health Research Involving Human Subjects, Part 3, Scope of Application (Chapter 1) state that the guidelines do not apply to studies conducted using only “anonymously processed information or unidentifiably-processed personal information which has already been created”. Given that the present study used only existing, anonymously processed information, it was considered to be outside the scope of the guidelines. In addition, this study posed no risks or disadvantages to the participants. The study was therefore not reviewed by an ethics review committee. Furthermore, none of the patients in the medical information database used in this study could be personally identified, and direct explanation of the study to the patients was not possible and no informed consent was therefore sought.

## RESULTS

### Study subjects

A total of 5,209,660 individuals with statutory insurance under health insurance policies were eligible for this 13-year data analysis from 2005 to 2017. The number of health-insured persons captured by the database was 277,585 in 2005, which increased to 3,827,283 in 2017, owing to an increase in the number of registered health insurance associations. However, throughout the study period, the type of health insurance remained the same (ie, employment-based health insurance only). The age distribution of our study population was slightly different from that of the general population. The proportion of 0–5-year-olds in our study population was 2 times higher than in the general population (0–5 years is the peak age of mumps onset). The sex distribution of our study population was almost the same as that of the general population (eTable 1). The average duration of health insurance subscription was 48.5 months.

### Annual incidence rate of mumps by age group

The incidence rate of mumps (per 100,000 person-years) varied across the age groups (Table 1). Most cases occurred in children aged 0–5 years every year, and the annual incidence rate of mumps in this age group ranged from 808 (95% CI, 772–846) in 2014 to 3,792 (95% CI, 3,571–4,023) in 2006. Among pediatric patients aged 6–15 years, the annual incidence rate of mumps ranged from 658 (95% CI, 632–684) in 2014 to 2,141 (95% CI, 2,007–2,281) in 2006. The annual incidence rate of mumps was approximately 10 times lower among adolescents/adults aged 16–45 years than in pediatric patients. It ranged from 21 (95% CI, 17–26) for 36–45-year-olds in 2013 to 149 (95% CI, 118–185) for 26–35-year-olds in 2005. The annual incidence rate of mumps was lowest in people aged 46–64 years, ranging from 0 (95% CI, 0–24) for 56–64-year-olds in 2005 to 23 (95% CI, 19–27) for 46–55-year-olds in 2016. Three outbreaks of mumps were noted in 2005–2006, 2010, and 2016 during the study period, mostly affecting the pediatric population aged 0–15 years. Trends were similar in each age group except for the 46–55 and 56–64 years age groups, which showed lower mumps case numbers (eFigure 1).

**Table 1.** Annual mumps incidence rates [95% CI] in males and females aged 0–64 years according to age group, based on the JMDC database cohort between 2005 and 2017^a^

Year	Annual analysis set, persons	Target subjects, person-years	Onset subjects, *n*	Mumps incidence rate^b^							

				All ages	0–5 years old^c^	6–15 years old	16–25 years old	26–35 years old	36–45 years old	46–55 years old	56–64 years old
2005	277,585	259,632	1,855	714 [682–748]	3,413 [3,200–3,636]	1,844 [1,717–1,977]	92 [63–129]	149 [118–185]	80 [58–107]	16 [5–37]	0 [0–24]
2006	295,132	272,672	2,170	796 [763–830]	3,792 [3,571–4,023]	2,141 [2,007–2,281]	103 [74–140]	129 [101–163]	106 [81–136]	19 [7–41]	0 [0–22]
2007	304,837	283,505	848	299 [279–320]	1,361 [1,230–1,502]	870 [787–960]	46 [28–71]	41 [26–62]	30 [18–47]	12 [3–30]	12 [1–42]
2008	417,298	388,251	1,288	332 [314–350]	1,654 [1,527–1,789]	932 [856–1,012]	47 [31–68]	55 [39–75]	39 [27–55]	16 [7–32]	7 [1–26]
2009	530,484	494,689	2,269	459 [440–478]	2,398 [2,260–2,542]	1,279 [1,200–1,361]	59 [43–79]	88 [69–110]	61 [47–78]	12 [5–23]	2 [0–14]
2010	679,050	637,513	3,525	553 [535–571]	2,485 [2,362–2,612]	1,846 [1,760–1,935]	87 [69–109]	105 [87–127]	81 [66–97]	15 [8–25]	9 [3–20]
2011	1,152,850	1,077,491	4,935	458 [445–471]	2,025 [1,938–2,114]	1,581 [1,520–1,644]	59 [48–73]	85 [72–99]	65 [55–76]	20 [14–29]	3 [1–10]
2012	1,619,148	1,518,257	4,428	292 [283–300]	1,273 [1,214–1,334]	1,035 [994–1,078]	46 [37–56]	65 [55–76]	34 [28–41]	13 [9–19]	7 [4–13]
2013	1,889,298	1,772,184	3,391	191 [185–198]	810 [767–854]	680 [649–713]	29 [23–37]	47 [39–56]	21 [17–26]	9 [6–14]	2 [1–6]
2014	2,791,170	2,612,857	4,803	184 [179–189]	808 [772–846]	658 [632–684]	33 [27–39]	38 [33–45]	24 [20–28]	10 [7–13]	5 [3–9]
2015	2,969,649	2,788,667	6,861	246 [240–252]	1,067 [1,027–1,108]	898 [869–928]	40 [34–47]	52 [45–59]	28 [24–33]	10 [8–13]	5 [3–9]
2016	3,727,843	3,504,480	16,664	476 [468–483]	1,920 [1,871–1,971]	1,949 [1,910–1,989]	80 [72–88]	116 [107–126]	72 [65–78]	23 [19–27]	14 [10–18]
2017	3,827,283	3,620,898	9,739	269 [264–274]	1,068 [1,031–1,106]	1,118 [1,089–1,147]	47 [42–54]	70 [64–78]	42 [37–47]	15 [12–18]	10 [7–14]

CI, confidence interval.

^a^Based on health insurance claims data from JMDC Inc.

^b^Per 100,000 target subjects (person year) in the corresponding age group.

^c^‘0’ indicates ≥3 months and <1 year old.

### Incidence of mumps by age group and sex

The overall incidence rate of mumps (per 100,000 person-years) during the study period was significantly higher in females than in males (344 vs 311, *P* < 0.001), with an average male/female ratio of 0.90 (Table 2). According to age group, the incidence was significantly higher in males than in females among 0–5-year-olds (*P* < 0.001) and 6–15-year-olds (*P* < 0.001), whereas in the age group 16 years and older, the incidence was significantly higher in females (16–25 years [*P* < 0.001], 26–35 years [*P* < 0.001], 36–45 years [*P* = 0.003], and 56–64 years [*P* = 0.007]).

**Table 2.** Overall mumps incidence rate [95% CI] during the study period in males and females (0–64 years old) in a JMDC database cohort, according to age and sex groups^a^

Sex	Target subjects (person)	Total observation period, person-years	Onset subjects, *n*	Mumps incidence rate^b^							

				All ages	0–5 years old^c^	6–15 years old	16–25 years old	26–35 years old	36–45 years old	46–55 years old	56–64 years old
All	5,209,660	21,007,982	68,307	325 [323–328]	1,552 [1,533–1,571]	1,186 [1,174–1,198]	52 [49–54]	71 [68–74]	44 [42–46]	15 [14–16]	8 [7–9]
Male	2,809,972	11,761,302	36,529	311 [307–314]	1,649 [1,622–1,676]	1,231 [1,214–1,249]	41 [38–44]	57 [54–60]	42 [39–44]	17 [15–19]	6 [5–8]
Female	2,399,688	9,246,680	31,778	344 [340–347]	1,449 [1,423–1,475]	1,138 [1,121–1,155]	66 [62–71]	92 [87–98]	48 [45–51]	13 [11–15]	10 [8–12]

^a^Based on health insurance claims data from JMDC Inc.

^b^Per 100,000 (person year).

^c^‘0’ indicates ≥3 months and <1 year old.

### Annual incidence of mumps-related complications

The peak incidences of mumps meningitis occurred in 2006, 2010, and 2016, corresponding to the peak incidences of mumps (Table 3 and eFigure 2A). However, the trends for orchitis, pancreatitis, and deafness did not correspond with the outbreaks of mumps (eFigure 2B). No cases of mumps oophoritis were noted during the study period.

**Table 3.** Annual incidence of mumps-related complications [95% CI] in males and females aged 0–64 years, based on the JMDC database cohort between 2005 and 2017^a^

Year	Mumps patients, *N*	Mumps-related complication^b^													

		All complications		Meningitis		Orchitis		Deafness^c^		Pancreatitis		Encephalitis		Other complications^d^	

		Onset patients, *n*	Incidence [95% CI]	Onset patients, *n*	Incidence [95% CI]	Onset patients *n*	Incidence [95% CI]	Onset patients, *n*	Incidence [95% CI]	Onset patients, *n*	Incidence [95% CI]	Onset patients, *n*	Incidence [95% CI]	Onset patients, *n*	Incidence [95% CI]
2005	1,855	16	8.6 [4.4–12.8]	10	5.4 [2.1–8.7]	5	2.7 [0.3–5.1]	0	0 [0.0–0.0]	0	0 [0.0–0.0]	0	0 [0.0–0.0]	1	0.5 [−0.5–1.6]
2006	2,170	19	8.8 [4.8–12.7]	14	6.5 [3.1–9.8]	4	1.8 [0–3.6]	1	0.5 [−0.4–1.4]	0	0 [0.0–0.0]	0	0 [0.0–0.0]	0	0 [0.0–0.0]
2007	848	7	8.3 [2.2–14.3]	3	3.5 [−0.5–7.5]	3	3.5 [−0.5–7.5]	0	0 [0.0–0.0]	0	0 [0.0–0.0]	0	0 [0.0–0.0]	1	1.2 [−1.1–3.5]
2008	1,288	8	6.2 [1.9–10.5]	4	3.1 [0.1–6.1]	2	1.6 [−0.6–3.7]	2	1.6 [−0.6–3.7]	1	0.8 [−0.7–2.3]	0	0 [0.0–0.0]	0	0 [0.0–0.0]
2009	2,269	22	9.7 [5.7–13.7]	9	4.0 [1.4–6.6]	9	4.0 [1.4–6.6]	3	1.3 [−0.2–2.8]	1	0.4 [−0.4–1.3]	0	0 [0.0–0.0]	0	0 [0.0–0.0]
2010	3,525	44	12.5 [8.8–16.1]	27	7.7 [4.8–10.5]	10	2.8 [1.1–4.6]	4	1.1 [0–2.2]	0	0 [0.0–0.0]	1	0.3 [−0.3–0.8]	2	0.6 [−0.2–1.4]
2011	4,935	50	10.1 [7.3–12.9]	29	5.9 [3.7–8.0]	14	2.8 [1.4–4.3]	5	1.0 [0.1–1.9]	4	0.8 [0–1.6]	0	0 [0.0–0.0]	1	0.2 [−0.2–0.6]
2012	4,428	52	11.7 [8.6–14.9]	22	5.0 [2.9–7.0]	13	2.9 [1.3–4.5]	10	2.3 [0.9–3.7]	2	0.5 [−0.2–1.1]	3	0.7 [−0.1–1.4]	3	0.7 [−0.1–1.4]
2013	3,391	38	11.2 [7.7–14.7]	17	5.0 [2.6–7.4]	10	2.9 [1.1–4.8]	3	0.9 [−0.1–1.9]	3	0.9 [−0.1–1.9]	5	1.5 [0.2–2.8]	0	0 [0.0–0.0]
2014	4,803	49	10.2 [7.4–13.0]	21	4.4 [2.5–6.2]	13	2.7 [1.2–4.2]	8	1.7 [0.5–2.8]	7	1.5 [0.4–2.5]	3	0.6 [−0.1–1.3]	1	0.2 [−0.2–0.6]
2015	6,861	75	10.9 [8.5–13.4]	41	6.0 [4.2–7.8]	19	2.8 [1.5–4.0]	8	1.2 [0.4–2.0]	3	0.4 [−0.1–0.9]	1	0.1 [−0.1–0.4]	4	0.6 [0–1.2]
2016	16,664	225	13.5 [11.7–15.3]	119	7.1 [5.9–8.4]	81	4.9 [3.8–5.9]	18	1.1 [0.6–1.6]	7	0.4 [0.1–0.7]	4	0.2 [0–0.5]	3	0.2 [0–0.4]
2017	9,739	127	13.0 [10.8–15.3]	55	5.6 [4.2–7.1]	45	4.6 [3.3–6.0]	21	2.2 [1.2–3.1]	4	0.4 [0–0.8]	2	0.2 [−0.1–0.5]	2	0.2 [−0.1–0.5]

CI, confidence interval.

^a^Based on health insurance claims data from JMDC Inc.

^b^Incidence per 1,000 mumps cases, respectively.

^c^Documented diagnosis of mumps deafness.

^d^Includes: arthritis, myocarditis, nephritis, polyneuropathy, hepatitis, and oophoritis.

### Incidence of mumps-related complications by age group and sex

The overall incidence of mumps-related complications (per 1,000 mumps cases) during the study period was 11.5 (95% CI, 10.7–12.4) (Table 4). The most common complications of mumps were meningitis, orchitis, and deafness. According to age group, the incidence (per 1,000 mumps cases) of mumps meningitis was highest (10.8) in the 26–35-year age group and lowest (4.2) in the 0–5-year age group (eFigure 3A). The incidence of mumps orchitis was highest (86.4) in the 26–35-year age group, followed by the 36–45-year (56.6) and 16–25-year (49.9) age groups (eFigure 3B). The incidence of mumps deafness was highest (19.0) in the 56–64-year age group, followed by the 46–55-year (12.8) and 26–35-year (7.1) age groups. The incidence of mumps pancreatitis was highest (2.6) in the 36–45-year age group, followed by the 26–35-year (2.1) and 16–25-year (2.0) age groups.

**Table 4.** Overall incidence of mumps-related complications [95% CI] during the study period in male and female mumps patients in a JMDC database cohort, according to age and sex^a^

Sex	Age group, years old	Mumps-related complication^b^																				

		All complications			Meningitis			Orchitis			Deafness^c^			Pancreatitis			Encephalitis			Other complications^d^		

		Mumps patients, *n*	Onset patients, *n*	Incidence [95% CI]	Mumps patients, *n*	Onset patients, *n*	Incidence [95% CI]	Mumps patients, *n*	Onset patients, *n*	Incidence [95% CI]	Mumps patients, *n*	Onset patients, *n*	Incidence [95% CI]	Mumps patients, *n*	Onset patients, *n*	Incidence [95% CI]	Mumps patients, *n*	Onset patients, *n*	Incidence [95% CI]	Mumps patients, *n*	Onset patients, *n*	Incidence [95% CI]
All	All ages	68,280	787	11.5 [10.7–12.4]	68,290	398	5.8 [5.3–6.4]	36,519	242	6.6 [5.8–7.5]	68,137	88	1.3 [1.0–1.6]	68,307	32	0.5 [0.3–0.7]	68,307	22	0.3 [0.2–0.5]	68,307	25	0.4 [0.2–0.5]
	0–5	26,050	132	5.1 [4.2–6.0]	26,050	110	4.2 [3.5–5.1]	14,231	2	0.1 [0.0–0.5]	26,013	7	0.3 [0.1–0.6]	26,056	6	0.2 [0.1–0.5]	26,056	5	0.2 [0.1–0.4]	26,056	2	0.1 [0.0–0.3]
	6–15	35,684	332	9.3 [8.3–10.4]	35,684	227	6.4 [5.6–7.2]	19,036	32	1.7 [1.2–2.4]	35,590	38	1.1 [0.8–1.5]	35,693	13	0.4 [0.2–0.6]	35,693	14	0.4 [0.2–0.7]	35,693	10	0.3 [0.1–0.5]
	16–25	1,529	60	39.2 [30.1–50.2]	1,531	13	8.5 [4.5–14.5]	701	35	49.9 [35.0–68.8]	1,523	8	5.3 [2.3–10.3]	1,531	3	2.0 [0.4–5.7]	1,531	1	0.7 [0.0–3.6]	1,531	3	2.0 [0.4–5.7]
	26–35	2,412	147	60.9 [51.7–71.2]	2,414	26	10.8 [7.0–15.7]	1,158	100	86.4 [70.8–104.0]	2,411	17	7.1 [4.1–11.3]	2,416	5	2.1 [0.7–4.8]	2,416	2	0.8 [0.1–3.0]	2,416	6	2.5 [0.9–5.4]
	36–45	1,898	85	44.8 [35.9–55.1]	1,904	19	10.0 [6.0–15.5]	972	55	56.6 [42.9–73.0]	1,896	8	4.2 [1.8–8.3]	1,904	5	2.6 [0.9–6.1]	1,904	0	0.0 [0.0–1.9]	1,904	3	1.6 [0.3–4.6]
	46–55	549	25	45.5 [29.7–66.5]	549	3	5.5 [1.1–15.9]	345	15	43.5 [24.5–70.7]	546	7	12.8 [5.2–26.2]	549	0	0.0 [0.0–6.7]	549	0	0.0 [0.0–6.7]	549	1	1.8 [0.0–10.1]
	56–64	158	6	38.0 [14.1–80.8]	158	0	0.0 [0.0–23.1]	76	3	39.5 [8.2–111.1]	158	3	19.0 [3.9–54.5]	158	0	0.0 [0.0–23.1]	158	0	0.0 [0.0–23.1]	158	0	0.0 [0.0–23.1]

Male	All ages	36,507	581	15.9 [14.7–17.3]	36,517	260	7.1 [6.3–8.0]	36,519	242	6.6 [5.8–7.5]	36,432	51	1.4 [1.0–1.8]	36,529	21	0.6 [0.4–0.9]	36,529	10	0.3 [0.1–0.5]	36,529	16	0.4 [0.3–0.7]
	0–5	14,228	80	5.6 [4.5–7.0]	14,228	66	4.6 [3.6–5.9]	14,231	2	0.1 [0.0–0.5]	14,206	3	0.2 [0.0–0.6]	14,231	6	0.4 [0.2–0.9]	14,231	1	0.1 [0.0–0.4]	14,231	2	0.1 [0.0–0.5]
	6–15	19,029	227	11.9 [10.4–13.6]	19,029	155	8.1 [6.9–9.5]	19,036	32	1.7 [1.2–2.4]	18,978	22	1.2 [0.7–1.8]	19,036	8	0.4 [0.2–0.8]	19,036	6	0.3 [0.1–0.7]	19,036	6	0.3 [0.1–0.7]
	16–25	701	49	69.9 [52.2–91.4]	703	7	10.0 [4.0–20.4]	701	35	49.9 [35.0–68.8]	697	5	7.2 [2.3–16.7]	703	2	2.8 [0.3–10.2]	703	1	1.4 [0.0–7.9]	703	2	2.8 [0.3–10.2]
	26–35	1,156	124	107.3 [90.0–126.5]	1,158	17	14.7 [8.6–23.4]	1,158	100	86.4 [70.8–104.0]	1,159	7	6.0 [2.4–12.4]	1,160	3	2.6 [0.5–7.5]	1,160	2	1.7 [0.2–6.2]	1,160	4	3.4 [0.9–8.8]
	36–45	972	72	74.1 [58.4–92.4]	978	12	12.3 [6.4–21.3]	972	55	56.6 [42.9–73.0]	974	6	6.2 [2.3–13.4]	978	2	2.0 [0.2–7.4]	978	0	0.0 [0.0–3.8]	978	1	1.0 [0.0–5.7]
	46–55	345	23	66.7 [42.7–98.4]	345	3	8.7 [1.8–25.2]	345	15	43.5 [24.5–70.7]	342	5	14.6 [4.8–33.8]	345	0	0.0 [0.0–10.6]	345	0	0.0 [0.0–10.6]	345	1	2.9 [0.1–16.0]
	56–64	76	6	78.9 [29.5–164.0]	76	0	0.0 [0.0–47.4]	76	3	39.5 [8.2–111.1]	76	3	39.5 [8.2–111.1]	76	0	0.0 [0.0–47.4]	76	0	0.0 [0.0–47.4]	76	0	0.0 [0.0–47.4]

Female	All ages	31,773	206	6.5 [5.6–7.4]	31,773	138	4.3 [3.7–5.1]	na	na	na	31,705	37	1.2 [0.8–1.6]	31,778	11	0.3 [0.2–0.6]	31,778	12	0.4 [0.2–0.7]	31,778	9	0.3 [0.1–0.5]
	0–5	11,822	52	4.4 [3.3–5.8]	11,822	44	3.7 [2.7–5.0]	na	na	na	11,807	4	0.3 [0.1–0.9]	11,825	0	0.0 [0.0–0.3]	11,825	4	0.3 [0.1–0.9]	11,825	0	0.0 [0.0–0.3]
	6–15	16,655	105	6.3 [5.2–7.6]	16,655	72	4.3 [3.4–5.4]	na	na	na	16,612	16	1.0 [0.6–1.6]	16,657	5	0.3 [0.1–0.7]	16,657	8	0.5 [0.2–0.9]	16,657	4	0.2 [0.1–0.6]
	16–25	828	11	13.3 [6.6–23.6]	828	6	7.2 [2.7–15.7]	na	na	na	826	3	3.6 [0.7–10.6]	828	1	1.2 [0.0–6.7]	828	0	0.0 [0.0–4.4]	828	1	1.2 [0.0–6.7]
	26–35	1,256	23	18.3 [11.6–27.4]	1,256	9	7.2 [3.3–13.6]	na	na	na	1,252	10	8.0 [3.8–14.6]	1,256	2	1.6 [0.2–5.7]	1,256	0	0.0 [0.0–2.9]	1,256	2	1.6 [0.2–5.7]
	36–45	926	13	14.0 [7.5–23.9]	926	7	7.6 [3.0–15.5]	na	na	na	922	2	2.2 [0.3–7.8]	926	3	3.2 [0.7–9.4]	926	0	0.0 [0.0–4.0]	926	2	2.2 [0.3–7.8]
	46–55	204	2	9.8 [1.2–35.0]	204	0	0.0 [0.0–17.9]	na	na	na	204	2	9.8 [1.2–35.0]	204	0	0.0 [0.0–17.9]	204	0	0.0 [0.0–17.9]	204	0	0.0 [0.0–17.9]
	56–64	82	0	0.0 [0.0–44.0]	82	0	0.0 [0.0–44.0]	na	na	na	82	0	0.0 [0.0–44.0]	82	0	0.0 [0.0–44.0]	82	0	0.0 [0.0–44.0]	82	0	0.0 [0.0–44.0]

^a^Based on health insurance claims data from JMDC Inc.

^b^Incidence per 1,000 mumps cases, respectively.

^c^Documented diagnosis of mumps deafness.

^d^Includes: arthritis, myocarditis, nephritis, polyneuropathy, hepatitis, and oophoritis.

na; not analyzable.

Comparing the two sexes, the overall incidence of mumps-related complications was higher in males than in females (15.9 vs 6.5, *P* < 0.001). Meningitis was also more common in males than in females (7.1 vs 4.3, *P* < 0.001). However, deafness, pancreatitis, and encephalitis showed similar incidences in males and females. The overall incidence of mumps-related complications did not differ between the sexes in the 0–5-year age group, but the incidence of mumps complications was higher in males than in females in all other age groups.

## DISCUSSION

This study aimed to estimate the incidence rates of mumps and its complications among Japanese people aged 0–64 years during the 13 years from 2005 to 2017, through an analysis of a health insurance reimbursement database. The analysis revealed three peaks of incidence indicating mumps outbreaks in 2005–2006, 2010, and 2016, which were temporally consistent with the published Infectious Diseases Weekly Report by the National Institute of Infectious Diseases.^6^ Our data showed that the mumps outbreaks mainly affected the pediatric population aged 0–15 years, suggesting an increased risk of infection in close-knit communities such as schools and nurseries after the loss of maternal antibodies. The incidence was higher in females than in males. Among adult females, the incidence was highest in the 26–35-year age group, suggesting possible transmission from infected children to female guardians. Yearly trends for each adult age group (except for 46–55 years and 56–64 years groups; in this study, reported mumps cases in 46–64-year-olds were limited) were generally aligned with the trends for pediatric groups. These findings also suggest that mumps epidemics in adults are closely related to epidemics in children.

The incidence of mumps-related complications seemed to increase over time. One possible reason is the increasing awareness of mumps. Recently, mumps deafness has become a prominent topic in Japanese medical societies. It is possible that this has increased awareness of mumps (and its complications), leading to more diagnoses of mumps complications.

The overall incidence of mumps-related complications was 2.5 times higher in males than in females, which could be explained by the high incidences of meningitis and orchitis in males. Regarding mumps meningitis, a previous study reported a higher incidence of mumps meningitis among children with natural mumps infection in males than in females,^12^ with an overall incidence of mumps meningitis of 13/1,051 (1.24%) among children with natural mumps infection.^12^ In our study, mumps meningitis was noted in 337 (0.55%) of 61,734 children aged 0–15 years, corresponding to an incidence of about half that mentioned above. This difference could be due to differences in sample sizes, study year, and vaccination statuses of the study populations.

Regarding mumps orchitis, a previous study reported a recent increase in mumps-related adult orchitis among males aged 15–24 years.^13^ The current study also found orchitis to be the most common complication of mumps. The incidence of mumps orchitis was highest in males aged 26–35 years, followed by males aged 36–46 years and 16–25 years, indicating that reproductive-age men were commonly affected. Given that mumps orchitis can cause male infertility, these results suggest the need for a mumps vaccine catch-up program to benefit these age groups, and also partly to address the falling birth rates. Davis et al reported that 13% of orchitis patients show lower fertility.^13^ The number of annual mumps patients is estimated as 431,000–1,356,000 in Japan.^14^ The present results showed no significant sex difference in mumps incidence. Additionally, the 26–45 years age group contained 5.8% of male mumps patients, suggesting an estimate of 12,499 to 39,324 annual male mumps patients in Japan. Furthermore, as our findings indicate that 7.3% of these have orchitis complications and a previous study indicated that 13% of orchitis patients show lower fertility,^13^ the number of mumps orchitis patients with lower fertility is likely to be 119 to 373 per year. As mumps-related impaired fertility is vaccine preventable, increasing the mumps vaccination rate is important from a fertility perspective.

Mumps deafness in most cases is a serious irreversible complication.^15^ Recently published Japanese data showed that the incidence of auditory disorders among mumps patients was 0.1–1%.^14^ In our study, mumps deafness was noted in 88 of 68,137 patients with mumps, with an incidence of 0.13% (1/774), which was consistent with the above published data.^14^ There were 45 cases of mumps deafness among mumps patients aged 0–15 years, accounting for about half of all cases of mumps deafness. However, the incidence of mumps deafness (per 1,000 mumps cases) was 0.7 among those aged 0–15 years and 6.6 among those aged 16–64 years, indicating an approximately 10 times higher risk in adolescents and adults than in children.

This study had some advantages, including the use of the JMDC Database, which contained long-term data for 13 years from 2005 to 2017 and covered approximately 4% of the entire Japanese population, with a broad age range of 0–64 years. Compared with data reported from pediatric sentinel sites, the data analyzed in this study thus allowed the determination of the mumps disease burden in a broader age range of people in Japan.

However, the study also had some limitations. Given that 30–40% of mumps virus infections are regarded as subclinical, the incidence of mumps cases might have been underestimated.^16^ In addition, the subjects in this study accounted for <5% of the entire Japanese population and did not include people older than 64 years, and the results may thus not be generalized to the entire Japanese population. The proportion in the JMDC Database of individuals aged 0–5 years, which is the peak age of mumps onset, is 2 times higher than in the general population. Therefore, it is possible that the incidence of mumps complications in those aged 0–64 years (all ages) was underestimated. In addition, the analyzed dataset did not contain information on the mumps vaccination status of the subjects, so the effects of this vaccination could not be taken into consideration. Although we had no information on subjects’ socioeconomic status, it is likely to be slightly biased, because the registered health insurance associations belong to large companies. Furthermore, the diagnosis coded at the health insurance reimbursement visit may have been wrong and may have differed from the actual final diagnosis.

In conclusion, this study revealed the disease burden due to mumps and its complications in Japan during 2005–2017. Mumps is a vaccine-preventable disease, and the findings suggest an urgent need for increasing the mumps vaccination coverage and immunizing citizens of all age groups against mumps, to reduce the burden due to mumps-related complications such as orchitis and deafness, which may otherwise lead to prolonged sequelae.

## APPENDIX A. SUPPLEMENTARY DATA

eTable 1. Comparison of age and sex distribution of study population and general population

eFigure 1. Annual changes in mumps incidence from 2005 to 2017 (per 100,000 person-years of target population). (A) All ages, 0–5 years, and 6–15 years; (B) 16–25 years, 26–35 years, 36–45 years, 46–55 years, and 56–64 years.

eFigure 2. Annual changes in mumps meningitis (A) and mumps orchitis (B) from 2005 to 2017

eFigure 3. Incidence of mumps meningitis (A) and mumps orchitis (B) by age group
