# Supplementary material for: Mumps-Related Disease Burden in Japan: Analysis of JMDC Health Insurance Reimbursement Data for 2005–2017
Source: J Epidemiol. 2021 Aug 5;31(8):464–70. doi: 10.2188/jea.JE20200048 (PMC8275443; doi:10.2188/jea.JE20200048)
Supplement: Supplementary file 1 [file je-31-464-s001.pdf]

**eTable 1.** Comparison of age and sex distribution of study population and general population

| Age | Study population | | General population[a] | |
|---|---|---|---|---|
| | population (1,000) | proportion (%) | population (1,000) | proportion (%) |
| Age | | | | |
| 0–5 | 655 | 12.6 | 5,734 | 6.4 |
| 6–15 | 589 | 11.3 | 10,582 | 11.7 |
| 16–25 | 921 | 17.7 | 12,374 | 13.7 |
| 26–35 | 924 | 17.7 | 13,192 | 14.6 |
| 36–45 | 945 | 18.1 | 14,794 | 16.4 |
| 46–55 | 712 | 13.7 | 19,986 | 22.1 |
| 56–64 | 464 | 8.9 | 13,618 | 15.1 |
| Sex | | | | |
| Male | 2,810 | 53.9 | 45,812 | 50.7 |
| Female | 2,400 | 46.1 | 44,472 | 49.3 |

[a]Estimated by the Cabinet Bureau of Statistics as of October 1, 2019.
https://www.stat.go.jp/data/jinsui/2019np/index.html
Accessed 28.04.20

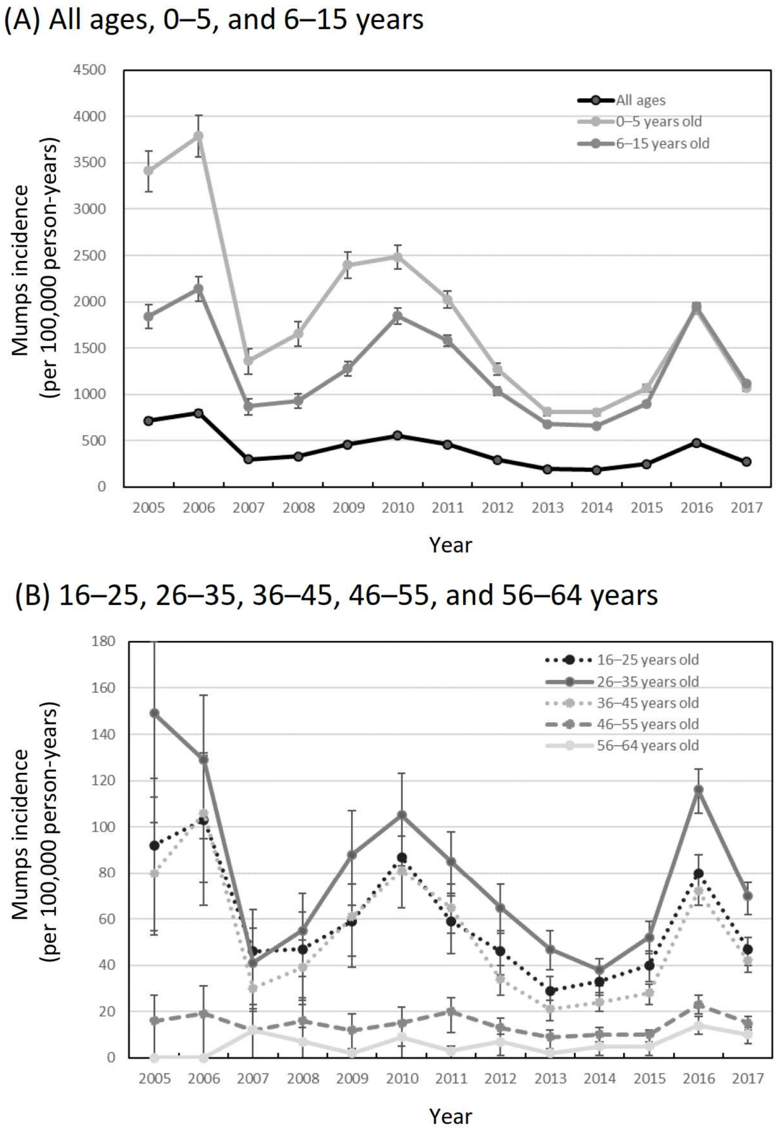


**eFigure 1.** Annual changes in mumps incidence from 2005 to 2017 (per 100,000 person-years of target population). (A) All ages, 0–5 years, and 6–15 years; (B) 16–25 years, 26–35 years, 36–45 years, 46–55 years, and 56–64 years.

(A) Mumps meningitis

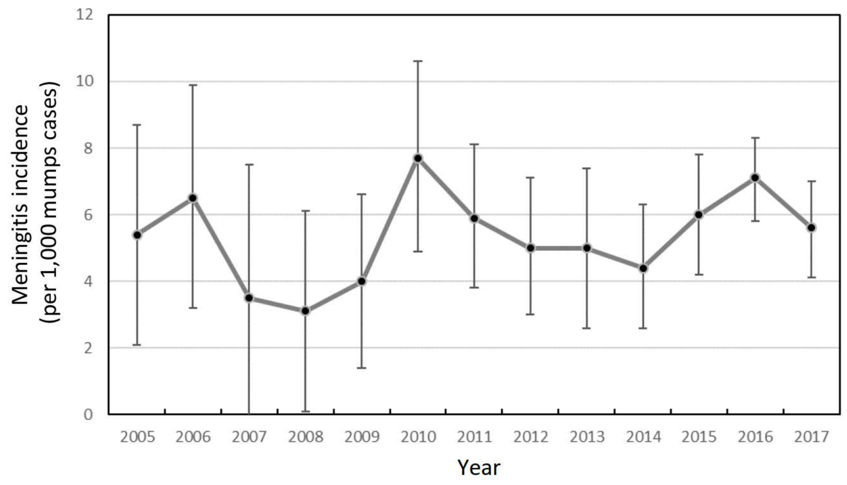


(B) Mumps orchitis

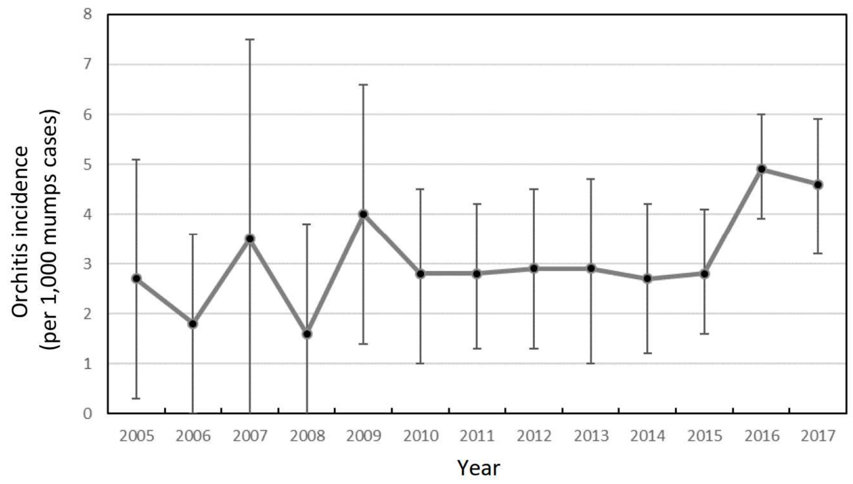


**eFigure 2.** Annual changes in mumps meningitis (A) and mumps orchitis (B) from 2005 to 2017

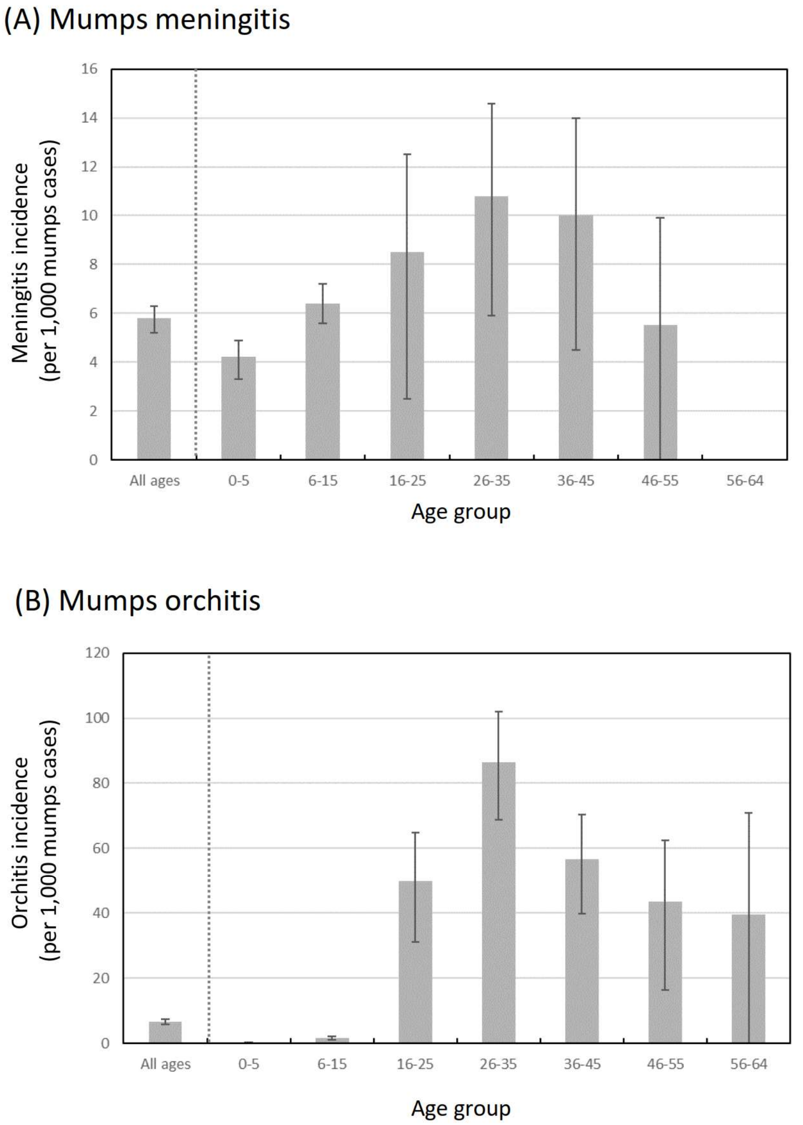


**eFigure 3.** Incidence of mumps meningitis (A) and mumps orchitis (B) by age group
